# Editorial: Addressing oral health disparities in maternal and child populations

**DOI:** 10.3389/froh.2026.1966251

**Published:** 2026-09-22

**Authors:** Abiola Adeniyi, Oyinkan Sofola

**Affiliations:** 1 School of Public and Global Affairs, Silberman College of Business, Fairleigh Dickinson University, Vancouver, BC, Canada; 2 Department of Periodontology and Community Dentistry, University of Lagos, Lagos, Akoka, Lagos, Nigeria

**Keywords:** access to oral healthcare, integrated oral healthcare, maternal and child oral health, oral health equity, universal oral health coverage

Oral diseases affect about 3.5 billion people globally (1, 2). Mothers and children in every region bear a disproportionate share of this burden. The consequences reach beyond the mouth. They affect health, social, and economic outcomes for families and communities. Fourteen articles in this Research Topic address the problem. They include prenatal care, community-based programs, provider training, national policy, and dental benefit analysis. Together, they show that closing these gaps requires coordinated action among healthcare providers, policymakers, researchers, and communities.

## Prenatal and maternal oral health

Three studies examine the period when maternal oral health first shapes a child's oral health outcomes. A pilot birth cohort study on prenatal total oral rehabilitation (PTOR) found that comprehensive dental treatment during pregnancy led to lasting improvements in maternal oral health. It also increased dental care use (3, Yang et al.). The study reported early signals that prenatal oral healthcare benefits infant oral health and reduces the risk of early childhood caries.

Whether mothers act on this potential depends on their beliefs about dental care during pregnancy. A study of 130 pregnant women in Southwestern Ontario found persistent misconceptions. Fewer than half understood that their oral health affects their child's health. Many also held unfounded fears about antibiotics, dental x-rays, and anesthetics. Many believed pregnancy inevitably leads to tooth loss. These beliefs likely keep people from seeking care when intervention matters most (Kamalabadi et al.).

Antenatal care providers face similar gaps. A survey of 459 obstetrician-gynecologists, nurses, and family physicians in Shanghai found suboptimal knowledge of oral health topics, particularly around medication and fluoride safety. Providers held a generally positive attitude toward integrating oral care into antenatal visits. Providers with prior training scored significantly higher, suggesting a clear solution: structured training for the professionals who most often care for pregnant women (Pan and Hu).

## Preparing providers to act on children's oral health

Two studies extend this training question to providers who work with children. A study of pediatric nurses caring for hospitalized children found significant gaps in oral health knowledge and practice, revealing an underused opportunity. The authors suggest that pediatric hospital care should routinely include oral health. Currently, it does not (Fletcher et al.).

A study of 50 non-dental primary care providers serving First Nations and Métis children in Manitoba identified four training needs: caries screening, use of a caries risk assessment tool, fluoride varnish application, and referral pathways. Providers suggested adopting a culturally safe, trauma-informed approach to care delivery. Preferred training format varied by geography. Urban providers preferred blended online and in-person training. Rural providers favored fully online delivery because of travel constraints. Together, these studies show that expanding preventive oral health care requires targeted investment in context-specific training (Olatosi et al.).

## Oral disease and children's quality of life

Two studies measure how dental disease affects children's daily lives. A study of 183 mother-child pairs in Chile found that dental caries accounted for over half of the relationship between maternal age and children's oral health-related quality of life. The finding suggests that caries prevention can help address equity gaps linked to maternal life-course factors (Castillo and Lancellotti). A national study of 892 Kuwaiti preschoolers found that 88.6% had early childhood caries (ECC). Nearly two-thirds of these cases showed extensive, untreated lesions. Caries experience strongly affected quality of life more than its severity. This indicates that even a high-income country with universal healthcare access can still face a significant and preventable disease burden (Alanzi et al.).

## Access, benefits, and policy

Four articles examine how systems and policies shape access. In the United States, an analysis of Medicaid-CHIP data found that children with public insurance have limited or no access to endodontic, oral surgery, or orthodontic specialists in about half of states. The gap is most severe for endodontic care, despite broad access to pediatric dentists (Cope et al.). Two companion studies evaluate Canada's Interim Canada Dental Benefit. An analysis of the first 18 months found that low-income families made up most approved applicants. The program allocated hundreds of millions of dollars to families in need. Geographic disparities in uptake remained (Goubran et al.). A follow-up analysis of the program's first year, focused on children under 12, shows that expanding affordability significantly improves access. Affordability alone, does not eliminate every barrier families face. Services must also be available where families live, particularly in rural and remote communities, and programs must address the accessibility, accommodation, and cultural fit of care, not just its cost (Schroth et al.).

## Community-based and culturally tailored approaches

Two studies examine how to integrate oral health promotion into trusted community settings. In Western Kenya, a proposed strategy integrates oral health promotion into Chamas for Change, a maternal health program built around microfinance. The strategy uses the WHO's Basic Package of Oral Care to reach mothers and children in areas without formal dental infrastructure (Adeniyi et al.). A qualitative study of immigrant parents in Norway examined an intervention with limited effectiveness. Parents valued native-language materials. Parents trusted public health nurses more than researchers as messengers. Cultural differences in norms around sugar and toothbrushing also shaped how guidance was received. Together, the two studies indicate that oral health promotion works best when it is part of trusted relationships and adapted to specific cultural contexts, rather than delivered as a generic message (Reda et al.).

## Prevention at the point of care

Two studies in Peru examine which preventive tools work, and whether they reach the children who need them. A randomized trial of 200 schoolchildren in Peru compared four topical fluoride formulations. 5% fluoride varnish maintained elevated salivary pH, a marker of protection against demineralization, longer than fluoride gels or standard toothpaste, suggesting that fluoride varnish offers superior protection for children in high-risk, high-altitude environments (Mamani-Cori et al.). A companion study found no association between access to dental services and use of adequately fluoridated toothpaste. Instead, toothpaste use varied by region, urban or rural residence, and household wealth. Improving access to dental care and expanding the availability of everyday preventive products are two separate problems. Each needs its own solution (Hadad-Arrascue and Garcés-Elías).

## Access is not enough

These 14 studies show one thing clearly: expanding access does not reliably translate into better oral health, and this should change how policymakers and funders think about closing this gap. Canada's dental benefit helped low-income families as intended, but it did not eliminate all the barrier they encountered (Goubran et al., Schroth et al.). Peruvian children with access to dental services were just as likely to use adequately fluoridated toothpaste as children without access (Hadad-Arrascue and Garcés-Elías). A country with universal healthcare access still faces one of the highest reported ECC burdens globally (Alanzi et al.). Access is necessary, but access alone is not enough. It must be combined with trust, training, and cultural fit. Without these, investment and appointments will not translate into disease prevention.

Funders and policymakers should stop viewing coverage expansion as the end goal. Instead, they need to invest in training, outreach, and trust-building to make coverage effective and accessible. Healthcare providers outside dentistry, including nurses, family physicians, obstetric teams, and community health workers, face a training barrier, not a lack of desire. Studies consistently show high interest alongside real gaps in preparation. Researchers should ask “for whom, delivered how, and trusted by whom” before asking “does the program work.” A fluoride varnish, a training format, or a benefit design that succeeds in one setting will not automatically succeed in another.

The WHO targets 2030 for universal oral health coverage (1). Based on this evidence, insurance and infrastructure alone will not meet that target. Training determines whether the providers families already see, nurses, family physicians, obstetric teams, and community health workers, can act on oral health rather than defer it to dentistry alone. Trust determines whether families use the services once they exist, since uptake follows the messenger as much as the message. Cultural adaptation determines whether guidance fits how a community actually lives, from language and norms around sugar and toothbrushing to who is seen as a credible source of health information. Coverage expansion creates the conditions for better oral health. It does not, on its own, produce it.
